# Electroacupuncture Mitigates TRPV1 Overexpression in the Central Nervous System Associated with Fibromyalgia in Mice

**DOI:** 10.3390/life14121605

**Published:** 2024-12-04

**Authors:** Doan Thi Ngoc Anh, Yi-Wen Lin

**Affiliations:** 1 College of Chinese Medicine, Graduate Institute of Acupuncture Science, China Medical University, Taichung 40402, Taiwan; dr.ngocanh0709@hotmail.com; 2 Chinese Medicine Research Center, China Medical University, Taichung 40402, Taiwan

**Keywords:** chronic pain, Fibromyalgia, Transient Receptor Potential Vanilloid 1, electroacupuncture, somatosensory cortex, medial prefrontal cortex

## Abstract

Background: Fibromyalgia (FM) is characterized by chronic pain, significantly affecting the quality of life and functional capabilities of patients. In addition to pain, patients may experience insomnia, chronic fatigue, depression, anxiety, and headaches, further complicating their overall well-being. The Transient Receptor Potential Vanilloid 1 (TRPV1) receptor responds to various noxious stimuli and plays a key role in regulating pain sensitivity and inflammation. Thus, targeting TRPV1 may provide analgesic and anti-inflammatory benefits. This study investigates the efficacy of electroacupuncture (EA) in alleviating chronic pain in FM through TRPV1 and its downstream molecules in the central nervous system (CNS). Methods: To model FM, we subjected mice to intermittent cold stress (ICS) for three days. The study comprised five rodent groups: Control (CON), ICS, ICS + EA, ICS + Sham EA, and ICS + KO (TRPV1 knockout mice). Results: Our findings revealed that ICS induced allodynia and hyperalgesia in mice by day four, persisting until day 21. EA at 2 Hz and TRPV1 KO significantly decreased both mechanical and thermal hypersensitivity (Withdrawal—Day 14: 2.43 ± 0.19 g; Day 21: 5.88 ± 0.47 g, n = 6, *p* < 0.05; Latency—Day 14: 2.77 ± 0.22 s; Day 21: 5.85 ± 0.41 s, n = 6, *p* < 0.05). In contrast, sham EA did not produce significant effects. Additionally, TRPV1 and several pain-related proteins were significantly elevated in the thalamus, somatosensory cortex (SSC), medial prefrontal cortex (mPFC), hippocampus, hypothalamus, cerebellum regions V (CB V), VI (CB VI) and VII (CB VII) after the ICS model. Both EA at the ST36 acupoint and TRPV1 KO mice showed diminished overexpression of pain-related proteins, with the sham EA group showing no significant changes compared to the ICS group. Conclusions: Chronic widespread pain was reduced by EA and TRPV1 KO, with the effects of EA on the TRPV1 pain pathway clearly evident in the CNS after 21 days.

## 1. Introduction

Fibromyalgia (FM) is defined as a long-term disorder characterized by widespread pain affecting muscles, bones, and soft tissues, typically accompanied by severe fatigue and sleep disruptions. Additional symptoms consist of cognitive impairment referred to as “fibro fog”, depression, migraines, irritable bowel syndrome, and pain in the pelvic region, jaw, and bladder [1]. In the eleventh revision of the International Statistical Classification of Diseases and Related Health Problems (ICD-11), FM is diagnosed under chronic widespread pain (MG30.01) and classified as a type of chronic primary pain [2]. The diagnosis of FM is based on criteria from the American College of Rheumatology, which include a widespread pain index, symptom severity reflecting weariness, poor sleep, and cognitive challenges, a minimum symptom duration of three months, and an unexplained pain origin [3]. FM symptoms can be mistaken for those of chronic fatigue syndrome, thyroid issues, or polymyalgia rheumatica; hence, paraclinical tests and X-rays are employed to rule them out because FM lacks distinct diagnostic indications [1,3]. Approximately 5% of the global population is affected by FM, with the majority of cases occurring in America, Europe, and a smaller proportion in Asia [4]. The disorder primarily impacts women (1:9 ratio) and manifests at any age [5,6]. Classified as nociplastic pain, FM is characterized by a lack of definitive neuropathic attributes and an association with altered nociceptive function without clear nociceptor activation [7].

FM is associated with the dysregulation of both excitatory and inhibitory neurotransmitters, including glutamate, norepinephrine, substance P, serotonin, opioids, GABA, and dopamine. Additionally, receptors such as 5HT-3, α2-adrenergic, μ-opioid, GABA-A, GABA-B, and TRPV1 play a crucial role in modulating pain signaling and regulating these neurotransmitters [8]. Transient Receptor Potential Vanilloid 1 (TRPV1) is a non-selective calcium channel. It can be activated by harmful stimuli, such as temperatures above 43 °C, pH changes, mechanical stimulation, or alterations in membrane permeability. Upon activation, TRPV1 triggers the release of substance P and glutamate [9,10]. Deletion of the TRPV1 gene suppressed thermal hyperalgesia in inflammatory models. TRPV1 agonists such as capsaicin and resiniferatoxin (RTX) induced rapid desensitization, leading to analgesic effects in experimental mice [11]. Although topical capsaicin cream offers short-term relief for FM pain, it may cause side effects in patients, including burning, stinging, or skin irritation [12]. Several TRPV1 antagonists have shown analgesic effects in the management of chronic FM pain [13,14].

Nishiyori et al. (2008) demonstrated that chronic allodynia and hyperalgesia are induced by the ICS model in mice [15]. This hypersensitivity involves the potentiation of TRPV1, although the underlying mechanisms remain unclear. Our previous study showed that TRPV1 activation is enhanced in the ICS model. Additionally, NGF signaling promotes an increase in TRPV1 receptor density in the membrane through the PI3K pathway [16]. Phosphorylation of PKA and PKC regulates TRPV1, with PKC increasing capsaicin-induced currents and reducing heat-induced allodynia [17]. Furthermore, upregulation of ERK, JNK, and P38 phosphorylation in the spinal cord has been detected after spinal nerve injury induction [18]. PKA-mediated phosphorylation of serine-133 on CREB occurs through the ERK pathway [19]. PKC and the PI3K/Akt pathway also influence the regulation of NF-kB activity [20]. Akt overexpression activates mTOR signaling in the hippocampus and cortex during neuropathic pain [21]. These complex molecular mechanisms highlight potential targets for therapeutic interventions that could address multiple pathways involved in neuropathic pain.

Nonpharmacological interventions, including cognitive behavioral therapy, exercise, relaxation therapy, electrical stimulation, psychotherapy, meditation, massage, and acupuncture, are recommended as first-line treatments to alleviate FM [22,23]. Acupuncture originated in China several thousand years ago and involves inserting fine needles to into acupoints [3]. *Deqi* is an essential sign of effective acupuncture, marked by sensations such as soreness, numbness, fullness, and heaviness, indicating successful activation of the meridian system. EA is a modern variation of acupuncture that integrates short-term manual acupuncture to elicit *deqi* with prolonged electrical stimulation. Systemic naloxone (2 mg/kg) blocked EA at 2–15 Hz, indicating that low-frequency electrical stimulation relies on the opioid system for analgesia [24]. Professor Han first provided an explanation for the scientific mechanism of acupuncture-induced release of neurotransmitters [25]. Wu et al. discovered that injecting capsaicin, a TRPV1 agonist, into ST36 (*Zusanli*) acupoints reproduced the analgesic effect observed with acupuncture [26]. In a recent study, EA and TRPV1 KO mice can reduce IL-17A levels in an ICS-induced FM-like pain model [27].

Evidence shows that acupuncture activates several brain regions, including the cerebral cortex and thalamus [28]. EA reduced TRPV1-pERK activation in the thalamus and SSC in acid saline-induced FM [29]. The hippocampal system is involved in EA analgesia and several chronic pain comorbidities, including fatigue, cognitive impairment, and depression [30,31]. In the mPFC, glucose metabolism was reduced by EA after chronic constriction injury induction [32]. The thalamus, SSC, mPFC, and hippocampus are involved in the ascending pathway. They receive sensory and motor sensory information from the peripheral nervous system (PNS). The hypothalamus and CB V, VI, and VII are more related to the descending pathway, which modulates nociception. The hypothalamus contains an ample supply of GABAergic neurons that regulate hyperalgesia and anxiety-like behavior [33]. The signal transmitted from EA to the hypothalamus has gained modern attention [34]. Consequently, investigation of both pain circuits might contribute to clarifying the EA analgesic mechanism.

While TRPV1 is involved in the pain signaling pathway of chronic FM pain, EA has demonstrated its therapeutic effects on FM symptoms. This study explores the mechanisms of EA through TRPV1 in a chronic ICS-induced FM-like pain model. Mechanical and thermal behavioral responses were examined, followed by the observation of the differential expression of TRPV1 and its downstream molecules in brain regions associated with both ascending and descending pain pathways across EA, TRPV1 gene KO, and other control groups. We suggest that both EA and KO decrease chronic hyperalgesia in FM via TRPV1 and its downstream signaling pathways.

## 2. Materials and Methods

### 2.1. Animal Research and Ethical Approval

The subjects of the present experiments were 8–12 weeks old female C57/BL6 wild-type mice acquired from BioLasc Taiwan Ltd. (Yilan, Taiwan), weighing 18–20 g. Female TRPV1 KO mice from Jackson Laboratory, Bar Harbor, ME, USA, were utilized for the study. Once the mice arrived, they were placed in Plexiglas cages with a 12-h alternating light and dark cycle environment, with the light phase beginning from 6 a.m. to 6 p.m., a normal room temperature of 25 °C, and 60 percent humidity. The researchers were blinded to the treatment section during the entire study.

Mice wererandomly assigned to one of five groups, including Control mice (CON), ICS-inducted FM mice (ICS), ICS treated by EA mice (ICS + EA), ICS treated by sham EA mice (ICS + Sham EA), and ICS in TRPV1 gene deletion (ICS + KO), with each group consisting of six mice.

China Medical University’s Institute of Animal Care and USE Committee, Taiwan, approved the usage of rodents in this research (Permit number: CMUIACUC-2022-424). In this study, the welfare of the mice was prioritized to minimize their distress. All mice were handled in accordance with the National Institutes of Health’s Guide for the Care and Use of Laboratory Animals.

### 2.2. Chronic FM Pain Induced by ICS

This study utilized the ICS in mice, developed by Nishiyori et al. (2008), which induces mechanical allodynia and thermal hyperalgesia lasting over 12 days and can be acutely alleviated by antidepressant treatment [28,35].

**Figure 1:**
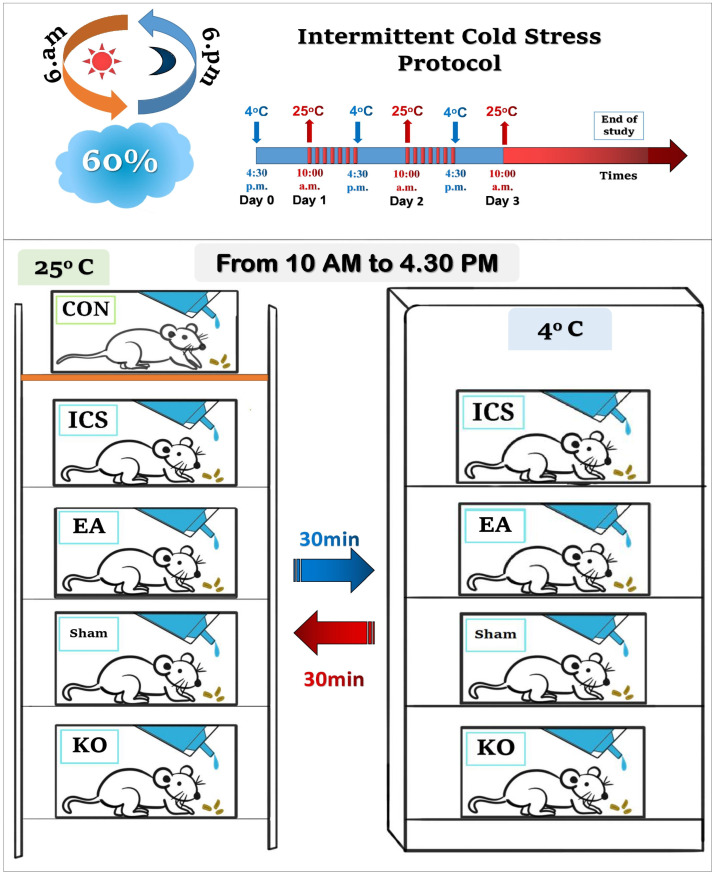
ICS protocol and condition of mice in five-group research. CON: control group; ICS: ICS group; EA: ICS + EA group; Sham: ICS + sham EA group; KO: ICS + TRPV1 KO group. All groups received ICS-induced FM-like pain, except the CON group.

Four groups of mice were placed in a 4 °C environment: ICS, ICS + EA, ICS + Sham EA, and ICS + KO, while the control group was maintained in a regular setting. At 10 a.m. the following day, these four groups were relocated to the outside environment at 25 °C for 30 min, then back to 4 °C for 30 min. This process was carried out until 4:30 p.m. for a total of 6 h, before they were repositioned again overnight from 4:30 p.m. during the first three days. After a week of acclimatization to their new environment, the mice underwent behavioral tests to determine their baseline mechanical and thermal thresholds, followed by the induction of chronic FM pain at 4:30 p.m. on day 0 (Figure 1).

### 2.3. EA Intervention

**Figure 2:**
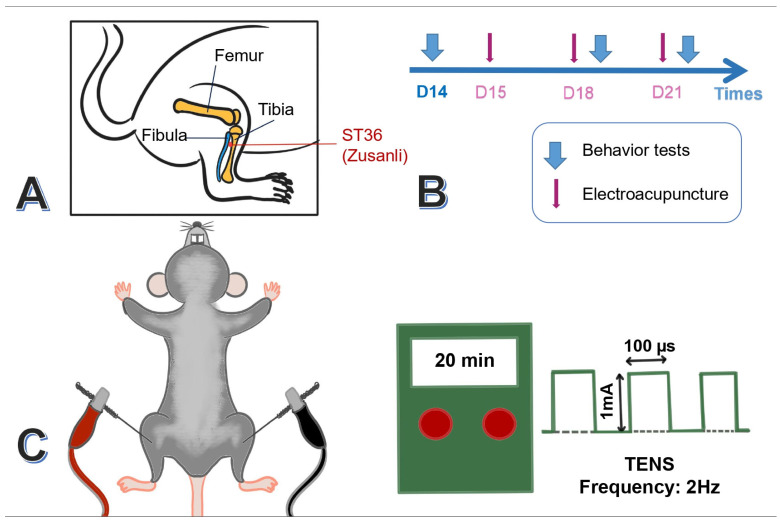
Application of bilateral EA at 2 Hz on the ST36 (Zusanli) acupoint in FM mice. (**A**) Anatomical location of ST36 in mice. (**B**) Timeline of EA administration on days 15, 18, and 21. (**C**) Electric stimulation protocol used in the ICS + EA group.

After being anesthetized with 4% isoflurane, the mice were placed in 1% gas via the head-stuffed tube connected to the chamber. Then, a pair of 0.5-inch-long acupuncture needles (32G, Yu Kuang Chem. Ind. Corp., Taipei, Taiwan) were inserted bilaterally at the mice’s ST36 acupoint (Zusanli). The murine ST36 was identified 3–4 mm lower than the patella, between the fibula and tibia, and on the anterior side of the anterior tibial muscle. In addition, a depth of 3–4 mm with 1 mA intensity, 2 Hz frequency, and 150 s width of the constant square pulse was performed for 20 min utilizing an electronic source from the machine (Trio 300 stimulator (Ito, Tokyo, Japan)) (Figure 2A,C). There was a mild twitch near the ST36 acupoint. Only the EA group received electric current, while the sham EA group was treated with sham acupuncture without electric current. Applications of EA took place on days 15, 18, and 21, prior to the behavioral tests (Figure 2B).

### 2.4. Mechanical and Thermal Behavior Tests

Both tests were performed seven times on days 0, 4, 7, 10, 14, 18, and 21 at the center of the plantar surface of the right hind paw. Mice were enclosed in Plexiglas boxes (9.5 × 11 × 6.5 cm) above a steel mesh in a dark, noiseless, room-temperature environment to keep them calm and adapted to their new surroundings.

The mechanical pain threshold was evaluated using the von Frey test, where a von Frey plastic tip was gently pressed against the middle of the mouse hind paw until the mouse suddenly withdrew the paw or licked it. The pressure at that moment was recorded as the threshold value, measured in grams. When mice were not sleeping, scratching, or grooming, the von Frey test was performed three times per session, each time 10 min apart, to ensure the mouse did not become accustomed to the test plastic tip and tolerate the electronic von Frey filament (IITC Life, Sciences, SERIES8, Model 390G, Burbank, Victory Blvd Woodland Hills, CA 91367, USA).

Then, the Hargreaves’ test, to measure the thermal response of mice, was performed. The mice were separated into various Plexiglass enclosures to restrict interactions. After 30 min of environmental habituation, the examination began. The IITC Plantar Analgesia Meter (IITC Life Sciences, SERIES8, Model 390G, Victory Blvd Woodland Hills, CA 91367, USA) determined the withdrawal delay of the mouse’s foot through radiant thermal light, which was placed underneath the tempered glass and slid across the surface to the exact middle of the plantar right hind paw. Each phase was also repeated three times for each rodent. The device was programmed to self-cut at 20 s to prevent harming the mice’ paws. The moment of foot withdrawal of the mice under the impact of heat was recorded in the unit of seconds. The outcomes were collected and calculated for mean values and standard errors.

### 2.5. Western Blotting Experiment (WB)

After completing behavioral response evaluations, rodents were euthanized on day 21. Before the procedure, the murine subjects were anesthetized via inhalation. In this study, we collected tissue samples from cerebral regions across both halves of the cerebrum, including the thalamus, SSC, mPFC, hippocampus, cerebellum V, VI, VII, and hypothalamus. The tissue was immediately placed on ice and kept at −80 degrees Celsius. During the experiment, manipulations were attentively carried out to minimize their misery.

Initially, protein homogenization was accomplished using 9 mL of double-distilled water, 1 mL of radioimmunoprecipitation (RIPA) lysis buffer (Fivephoton Biochemicals, RIPA-50, San Diego, CA 92117, USA), 100 μL of protease inhibitor (Bionovas, FC0070-0001, Taipei, Taiwan), and 100 μL of phosphatase inhibitor (Bionovas, FC0050-0001, Taipei, Taiwan). About 10 μL to 20 μL (depending on the tissue sample size) of extracted protein was electrophoretically loaded in an 8% sodium dodecyl sulfate (SDS)-Tris-glycine gel. A current electrophoresis power supply (PowerPac, Singapore) was used to run gels, which were separated into two sections lasting 1 h 30 min (at 100 V) and 50 min (at 50 V), respectively. Then, proteins on gel were transferred onto the Polyvinylidene fluoride (PVDF) membrane utilizing a semi-dry transfer machine (Trans-Blot SD Cell, San Diego, CA, USA) for 45 min (at 15 V). The transferred membranes were quickly cleaned with phosphate-buffered saline Tween (PBST with 0.05% Tween 20) and blocked with bovine serum albumin at 4 °C for 10 min.

Subsequently, membranes were incubated in the following primary antibodies (1:1000): anti-TRPV1 (~95 kDa, Alomone, ACC-030, Jerusalem, Israel), anti-pPI3K (~130 kDa, Abcam, ab154598, Cambridge, UK), anti-pAkt (~65 kDa, Thermo Fisher Scientific, 44-621G, Washington, DC, USA), anti-pJNK (~65 kDa, Millipore, 16-293, Burlington, MA, USA), anti-pmTOR (~180 kDa, Millipore, 09-213, Burlington, MA, USA), anti-pERK1/2 (~42 kDa, Abcam, ab138482, Cambridge, UK), anti-pP38 (~41 kDa, Thermo Fisher Scientific, 44-684G, Washington, DC, USA), anti-pNF-κB (~65 kDa, Abcam, ab86299, Cambridge, UK), anti-pCREB (~43 kDa, Cell Signaling Technology, 9198, Burlington, MA, USA), anti-pPKC (~82 kDa, Santa Cruz Biotechnology, SC-12355, San Diego, CA, USA), anti-pPKAII (~40 kDa, Santa Cruz Biotechnology, SC-12905, CA, USA), and anti-tubulin (~55 kDa, Santa Cruz Biotechnology, SC-5286, CA, USA), in PBST containing bovine serum albumin (BSA) 1% overnight at a temperature of 4 °C.

After three washes with PBST, secondary antibodies (1:5000) were applied to incubate with the membrane for a period of two hours at 25 °C, including peroxidase-conjugated goat anti-rabbit antibody and goat anti-mouse antibody (Jackson Immuno Research Laboratory, Ely, UK). The protein bands on the membranes were visualized using an enhanced chemiluminescence substrate kit (PIERCE) and by means of Celvin S (Biostep, Burkhardtsdorf, Germany). The NIH ImageJ software 1.53e (Bethesda, MD, USA) was employed to determine the image densities for certain protein bands. α-Tubulin was used as the internal control.

### 2.6. Statistical Analysis

This study compares five groups of mice in terms of behavioral measurements and WB analysis. The collected information was statistically analyzed using the Lab Origins program. All statistical data are presented as the mean ± standard error (SEM).

The average results of three applications on each mouse during the mechanical and thermal tests were used to calculate the mean value and standard error (SE) of the behavioral measurement. For the Western blot analysis, α-tubulin was used as an internal control protein. After determining the image densities, each protein’s WB value was calculated as the percentage of its intensity relative to tubulin intensity. For both behavioral and WB results, statistically significant differences among the five experimental groups were determined using repeated one-way analysis of variance (one-way ANOVA). Repeated One-way ANOVA yields significant results between the five groups, * compared to CON or # compared to the ICS group, followed by Tukey’s post hoc test. *p* < 0.05, 0.01, or 0.001 were considered as significant.

## 3. Results

### 3.1. ICS-Induced Mechanical Allodynia and Thermal Hyperalgesia in Mice and Assessing the Efficacy of EA at ST36 for Chronic FM Pain

#### 3.1.1. Von Frey Test

**Figure 3:**
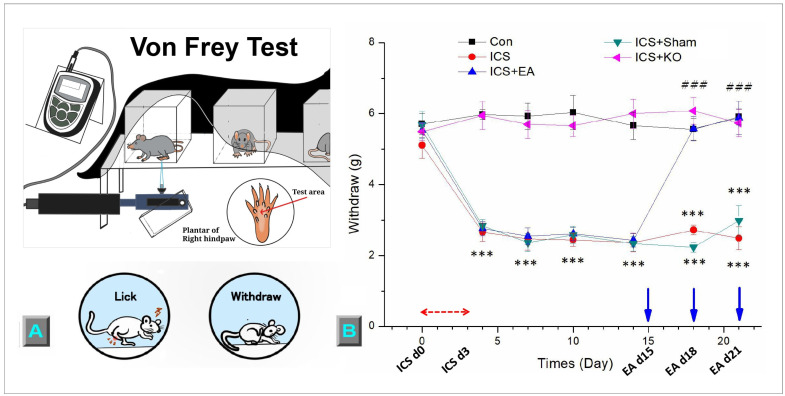
This diagram shows the mechanical threshold of mice in five groups. (**A**) The process involved in the mechanical test; (**B**) von Frey test measured the withdrawal threshold (unit: grams = g). The study involved five groups: CON, ICS, ICS + EA, ICS + Sham, and ICS + KO. The ICS model was applied during the first three days (ICS d0 to d3), and EA or sham EA were administered on days 15, 18, and 21, prior to the von Frey test. Each mouse was measured three times, with 10-min intervals between measurements. Statistical differences were analyzed using one-way ANOVA and Tukey’s post hoc test. *** *p* < 0.001, compared to the CON group; ### *p* < 0.001, compared to the ICS group. Red arrows mean ICS induction. Blue arrows mean EA treatment.

The von Frey test was used to assess mechanical hyperalgesia in mice before and after exposure to ICS and the intervention (Figure 3A). Figure 3B illustrates the timeline of the entire experiment on the x-axis. The withdrawal threshold was measured in all groups of mice to evaluate the analgesic effect of EA. The mice received ICS experienced a significant reduction in their mechanical threshold levels (Figure 3B, red line, D4 = 2.66 ± 0.26 g, *** *p* < 0.001, n = 6), except for the ICS + KO mice (Figure 3B, pink line, D4 = 5.95 ± 0.39 g, * *p* < 0.05, n = 6). Chronic FM pain induction was confirmed by the persistence of the hypersensitive response in the ICS group, compared to the CON group (Figure 3B, red line, D21 = 2.49 ± 0.32 g vs. black line, D21 = 5.91 ± 0.23 g, *** *p* < 0.001, n = 6). EA at 2 Hz remarkably mitigated mechanical hyperalgesia after the second and third interventions, compared to the ICS group (Figure 3B, blue line, D18 = 5.57 ± 0.33 g, D21 = 5.89 ± 0.47 g, ### *p* < 0.001, n = 6). Additionally, the TRPV1 KO group also showed attenuation of mechanical hyperalgesia after ICS induction (Figure 3B, pink line, D21 = 5.75 ± 0.4 g, ### *p* < 0.001, n = 6). However, the sham EA did not change this phenomenon after three sessions (Figure 3B, green line, D18 = 2.23 ± 0.13 g, D21 = 2.98 ± 0.44 g, *** *p* < 0.001, n = 6).

#### 3.1.2. Hargreaves’ Test

**Figure 4:**
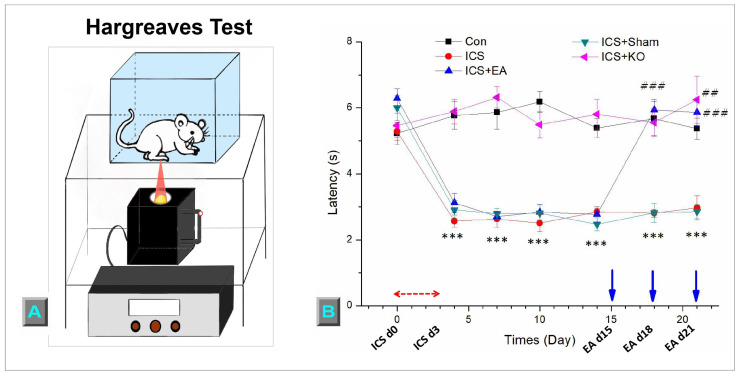
This diagram illustrates the thermal sensitivity of mice across five groups. (**A**) The withdrawal threshold was assessed using the Hargreaves test (seconds = s), (**B**) The figure illustrates the procedure of the thermal test. The study involved five groups: CON, ICS, ICS + EA, ICS + Sham, and ICS + KO. The ICS model was applied during the first three days (ICS d0, d3), and EA was administered on days 15, 18, and 21 (EA d15, d18, d21), prior to the von Frey test. Each mouse was measured three times, with 10-min intervals between measurements. Statistical differences were analyzed using one-way ANOVA and Tukey’s post hoc test. *** *p* < 0.001, compared to the CON group; ### *p* < 0.001, ## *p* < 0.01, compared to the ICS group. Red arrows mean ICS induction. Blue arrows mean EA treatment.

Hargreaves’ test was carried out at the same time as the von Frey test. After the mechanical measurement, the mice were given time to rest and calm down to avoid stress. Then, the Hargreaves’ test was used to assess the latency threshold at the same area of the mouse paw as the von Frey test (Figure 4A). Each application was administered three times for each mouse. The sharp reduction in the latency threshold in the ICS group indicates that the ICS model induced thermal hyperalgesia (Figure 4B, red line, D0 = 5.29 ± 0.27, D4 = 2.57 ± 0.18, *** *p* < 0.001, n = 6). Chronic hypersensitivity is clearly demonstrated through the low latency threshold of ICS mice over 21 days, compared to the CON group (Figure 4B, red line, D21 = 2.97 ± 0.37 s vs. black line, D21 = 5.37 ± 0.33 s, *** *p* < 0.001, n = 6). Low-frequency EA treatment suppressed thermal hyperalgesia by significantly elevating the latency level (Figure 4B, blue line, D18 = 5.93 ± 0.32 s; D21 = 5.86 ± 0.41 s, ### *p* < 0.001). Furthermore, ICS + KO mice showed the analgesic effect on eliciting thermal hyperalgesia (Figure 4B, pink line, D4 = 5.89 ± 0.37 s; D21 = 6.25 ± 0.72, ## *p* < 0.01). In contrast, the ICS + sham EA group did not show this effect (Figure 4B, green line, D18 = 2.82 ± 0.28 s; D21 = 2.86 ± 0.21, *** *p* < 0.001).

### 3.2. EA Remarkably Alleviated TRPV1 and Its Downstream Pain Signaling Pathway in the CNS of FM Mice

#### 3.2.1. The Expression of TRPV1 and Related Proteins in Thalamus and SSC

The earlier behavioral tests confirmed the successful induction of chronic FM pain at the peripheral nociceptive level. WB was used to investigate the TRPV1 signaling pathway involved in the analgesic effect of EA in the CNS. Since the thalamus and SSC play important roles in chronic FM pain, we assessed the expression of TRPV1 and associated proteins in the thalamus and SSC of mice (Table 1). The CON group was established as 100% and served as a reference. Significance values were reported with the standard error of the mean (SEM), and *p*-values of 0.01 and 0.001 were deemed statistically significant (Table 1 and Table 2).

**Figure 5:**
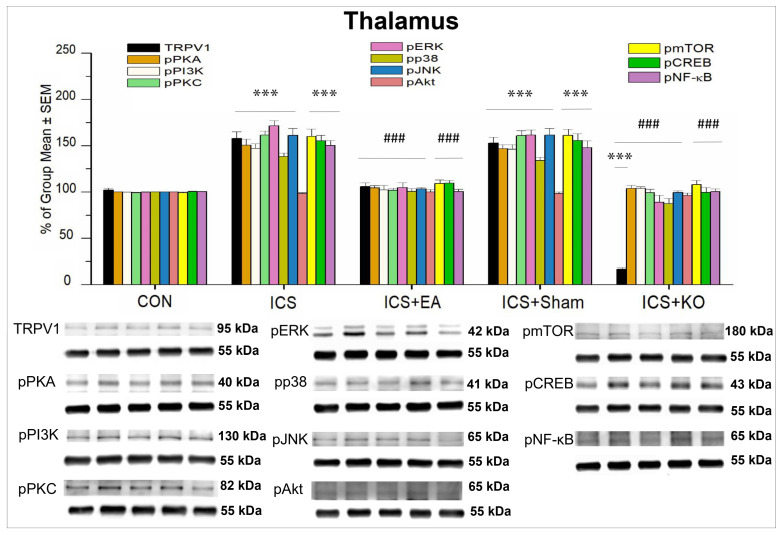
Percentage changes in the expression of TRPV1 and associated molecules in the thalamus of mice (%). The control group was set as 100% and served as a reference. The WB analysis included five lanes for each protein: CON, ICS, ICS + EA, ICS + Sham, and ICS + KO. The proteins observed were TRPV1, pPKA, pPI3K, pPKC, pAkt, pmTOR, pERK, pP38, pJNK, pCREB, and pNF-κB. α-Tubulin was used as an internal control protein. Statistical differences were analyzed using one-way ANOVA, followed by Tukey’s post hoc test. *** *p* < 0.001, compared to the CON group; ### *p* < 0.001, compared to the ICS group. n = 6.

In the thalamus, TRPV1 and its downstream molecules—pPKA, pPI3K, pPKC, pERK, pP38, pJNK, pAkt, pmTOR, pCREB, and pNFκB—were found in CON mice (Table 1). The results showed that thalamic sensitization manifested similarly to peripheral nociceptive hyperalgesia. WB analysis revealed alterations in the expression levels of TRPV1 and its downstream molecules (Table 1). Expression of these proteins was detected in the thalamus of CON mice. There was a notable upregulation in most of the protein expressions studied in the ICS group (Figure 5, *** *p* < 0.001, n = 6), except for pAkt. EA 2 Hz effectively reduced this overexpression after three interventions (Figure 5, ### *p* < 0.001, n = 6). The increase in these proteins was significantly reduced by KO treatment. (Figure 5, ### *p* < 0.001, n = 6). Additionally, TRPV1 expression in KO mice was notably lower compared to the other groups (Figure 5, *** *p* < 0.001, ### *p* < 0.001, n = 6). However, this attenuation was not seen in the sham EA group (Figure 5, *** *p* < 0.001, n = 6). Notably, pAkt percentage did not differ significantly between groups.

**Figure 6:**
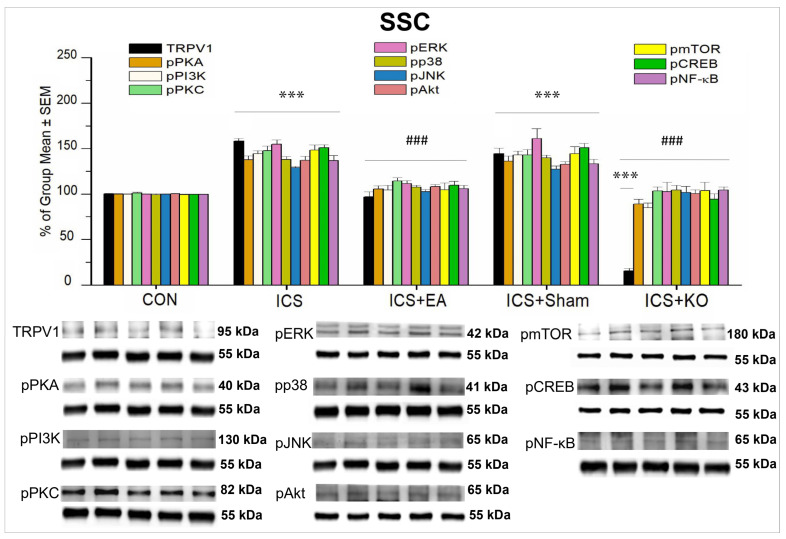
Percentage changes in the expression of TRPV1 and associated molecules in the SSC of mice (%). The control group was set as 100% and served as a reference. The WB analysis included five lanes for each protein: CON, ICS, ICS + EA, ICS + Sham, and ICS + KO. The proteins observed were TRPV1, pPKA, pPI3K, pPKC, pAkt, pmTOR, pERK, pP38, pJNK, pCREB, and pNF-κB. α-Tubulin was used as an internal control protein. Statistical differences were analyzed using one-way ANOVA, followed by Tukey’s post hoc test. *** *p* < 0.001, compared to the CON group; ### *p* < 0.001, compared to the ICS group. n = 6.

Our study showed that TRPV1 and its downstream molecules were expressed on SSC. The significant increase in the percentage of TRPV1, pPKA, pPI3K, pPKC, pERK, pP38, pJNK, pAkt, pmTOR, pCREB, and pNF-κB after FM was seen in the ICS group (Figure 6, *** *p* < 0.001, n = 6). EA and KO intervention effectively reduced this overexpression (Figure 6, ### *p* < 0.001 vs. ICS, n = 6), but not sham EA (Figure 6, *** *p* < 0.001 vs. CON, n = 6). This indicates that TRPV1 and its downstream molecules play a vital role in pain signaling transmission and are involved in the EA effect in the SSC. Similar to the thalamus, TRPV1 expression in KO mice was significantly lower than that of other proteins (Figure 6, *** *p* < 0.001, n = 6).

#### 3.2.2. TRPV1 and Its Related Proteins in the mPFC and Hippocampus

The brain regions associated with the ascending pain pathway also include the mPFC and hippocampus. The percentages of TRPV1 and its related proteins for these two brain regions are illustrated in Table 2.

**Figure 7:**
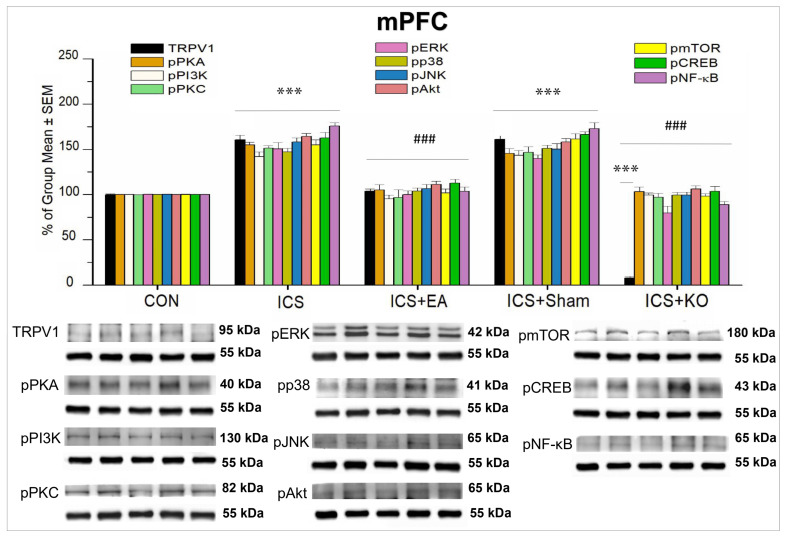
Percentage changes in the expression of TRPV1 and associated molecules in the mPFC of mice (%). The control group was set as 100% and served as a reference. The WB analysis included five lanes for each protein: CON, ICS, ICS + EA, ICS + Sham, and ICS + KO. The proteins observed were TRPV1, pPKA, pPI3K, pPKC, pAkt, pmTOR, pERK, pP38, pJNK, pCREB, and pNF-κB. α-Tubulin was used as an internal control protein. Statistical differences were analyzed using one-way ANOVA, followed by Tukey’s post hoc test. *** *p* < 0.001, compared to the CON group; ### *p* < 0.001, compared to the ICS group. n = 6.

In the mPFC, we further determined the distribution of TRPV1 and its downstream signaling pathways in CON mice. The percentage of all analyzed proteins in the FM-induced group was significantly higher than that in CON (Figure 7, *** *p* < 0.001, n = 6). These protein activations were alleviated by EA treatment at ST36 acupoints (Figure 7, ### *p* < 0.001, n = 6). Additionally, KO mice showed an inhibitory effect on TRPV1 activation, resulting in a reduction in the activity of the remaining proteins (Figure 7, ### *p* < 0.001, n = 6,). In contrast, sham EA did not change these proteins’ excessive activity (Figure 7, *** *p* < 0.001, n = 6). TRPV1 expression in the KO group was similarly low compared to the CON group (Figure 7, *** *p* < 0.001, n = 6).

We also studied the hippocampus in mice, which is involved in both ascending and descending pain pathways. Our analysis revealed that TRPV1, pPKA, pPI3K, pPKC, pAkt, pmTOR, pERK, pP38, pJNK, pCREB, and pNF-κB were distributed in the hippocampus of CON mice. Nonetheless, several proteins, including pPKA, pPKC, pERK, pP38, pAkt, and pCREB, showed no significant differences across the five subject groups (Table 2).

**Figure 8:**
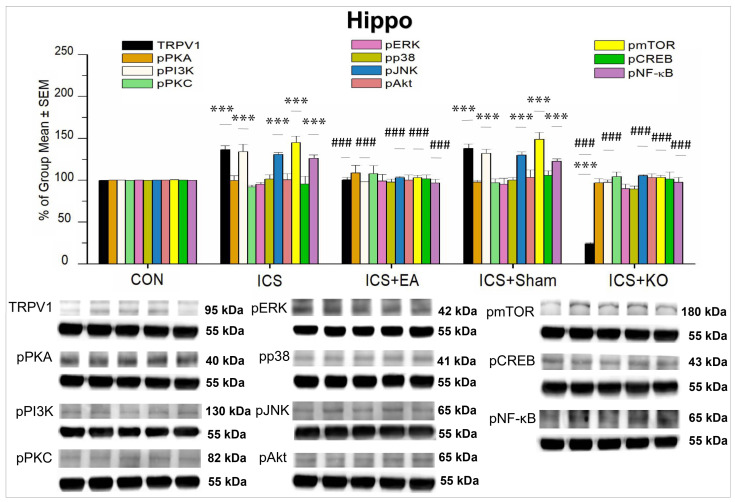
Percentage changes in the expression of TRPV1 and associated molecules in the hippocampus of mice (%). The control group was set as 100% and served as a reference. The WB analysis included five lanes for each protein: CON, ICS, ICS + EA, ICS + Sham, and ICS + KO. The proteins observed were TRPV1, pPKA, pPI3K, pPKC, pAkt, pmTOR, pERK, pP38, pJNK, pCREB, and pNF-κB. α-Tubulin was used as an internal control protein. Statistical differences were analyzed using one-way ANOVA, followed by Tukey’s post hoc test. *** *p* < 0.001, compared to the CON group; ### *p* < 0.001, compared to the ICS group. n = 6.

On the other hand, TRPV1, pPI3K, pJNK, pmTOR, and pNF-κB proteins in both the ICS and ICS + Sham groups concurrently increased after FM induction, but this rise was attenuated by EA treatment (Figure 8, *** *p* < 0.001 vs. CON, ### *p* < 0.001 vs. ICS, n = 6). KO treatment demonstrated its significant effect by downregulating the overexpression of these proteins, compared with the ICS group (Figure 8, ### *p* < 0.001, n = 6). The percentage of TRPV1 in the KO group was considerably lower than that in the CON group (Figure 8, ### *p* < 0.001, n = 6). The differential expression of these proteins in the hippocampus indicates that they may have a location-specific function.

#### 3.2.3. Role of TRPV1 and Related Proteins in the Descending Pathways of CB V and CB VI

**Figure 9:**
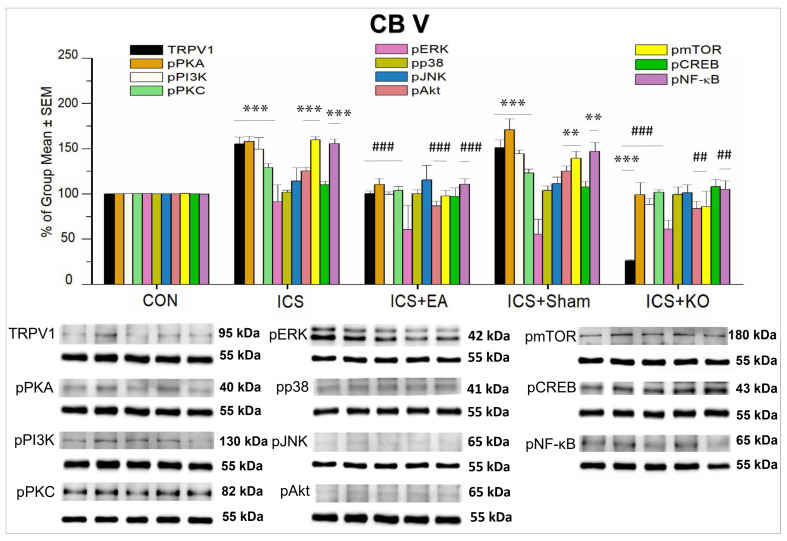
Percentage changes in the expression of TRPV1 and associated molecules in the CB V of mice (%). The control group was set as 100% and served as a reference. The WB analysis included five lanes for each protein: CON, ICS, ICS + EA, ICS + Sham, and ICS + KO. The proteins observed were TRPV1, pPKA, pPI3K, pPKC, pAkt, pmTOR, pERK, pP38, pJNK, pCREB, and pNF-κB. α-Tubulin was used as an internal control protein. Statistical differences were analyzed using one-way ANOVA, followed by Tukey’s post hoc test. *** *p* < 0.001, ** *p* < 0.01, compared to the CON group; ### *p* < 0.001, ## *p* < 0.01, compared to the ICS group. n = 6.

Descending pain pathways also contribute to pain modulation, either amplifying or inhibiting pain signals. Therefore, we used WB to study the expression of the aforementioned proteins in CB V and VI. Data showed that there were expressions of TRPV1 and its downstream molecules in CB V. The outcomes of pERK, pJNK, pP38, and pCREB in CB V revealed no significant differences in percentage among the research groups (Table 3). This implied that in CB V, these proteins were not crucial for TRPV1-related pain signaling in FM pain. However, TRPV1, pPKA, pPI3K, and pPKC were found to increase after ICS induction (Figure 9, *** *p* < 0.001, n = 6). EA and KO treatments reliably reduced TRPV1, pPKA, pPI3K, and pPKC increases (Figure 9, ### *p* < 0.001, n = 6), but this was not the case in sham mice (Figure 9, *** *p* < 0.001, n = 6). TRPV1 gene deletion caused a dramatic downregulation in its expression (Figure 9, *** *p* < 0.001, n = 6). Similarly, increased pAkt, pmTOR, and pNF-κB were also augmented after FM induction (Figure 9, *** *p* < 0.001, n = 6). However, this increase was further reversed by EA (Figure 9, *** *p* < 0.001, n = 6), but not sham EA (Figure 9, ** *p* < 0.01, n = 6). KO treatment effectively reduced this overexpression induced by FM (Figure 9, ## *p* < 0.01, n = 6).

**Figure 10:**
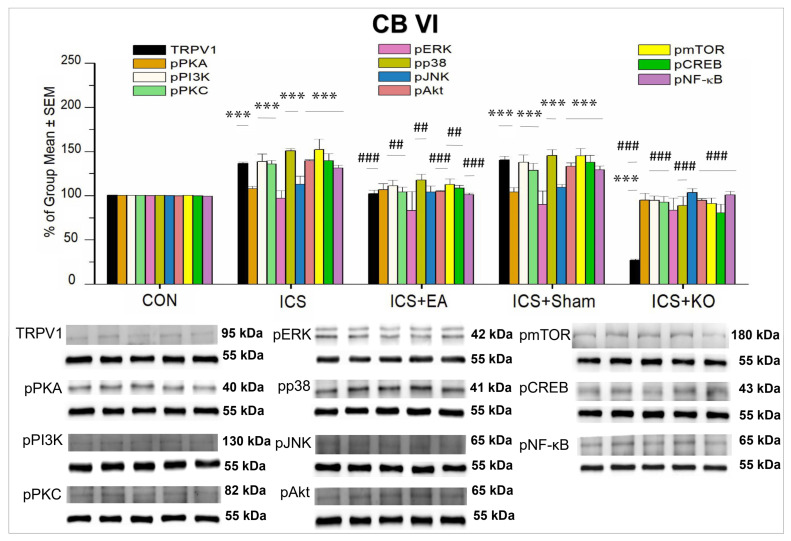
Percentage changes in the expression of TRPV1 and associated molecules in the CB VI of mice (%). The control group was set as 100% and served as a reference. The WB analysis included five lanes for each protein: CON, ICS, ICS + EA, ICS + Sham, and ICS + KO. The proteins observed were TRPV1, pPKA, pPI3K, pPKC, pAkt, pmTOR, pERK, pP38, pJNK, pCREB, and pNF-κB. α-Tubulin was used as an internal control protein. Statistical differences were analyzed using one-way ANOVA, followed by Tukey’s post hoc test. *** *p* < 0.001, compared to the CON group; ### *p* < 0.001, ## *p* < 0.01, compared to the ICS group. n = 6.

Similar to CB V, all proteins were distributed in the CB VI of CON mice. In CB VI, we identified an association between central sensitization and peripheral nociceptive signaling through TRPV1 and various proteins in the pain signaling pathway, including pPI3K, pPKC, pP38, pAkt, pmTOR, pCREB, and pNF-κB (Table 3). We verified that TRPV1, pAkt, and pNF-kB were significantly potentiated in ICS mice (Figure 10, *** *p* < 0.001, n = 6). This potentiation was then reversed by EA and KO treatments (Figure 10, ### *p* < 0.001, n = 6), but not sham EA (Figure 10, *** *p* < 0.001, n = 6). TRPV1 levels were noticeably lower in KO mice than in CON mice (Figure 10, *** *p* < 0.001, n = 6). Furthermore, pPI3K, pP38, and pmTOR were measured as TRPV1 downstream molecules and simultaneously increased after inducing FM (Figure 10, *** *p* < 0.001, n = 6). They were further abrogated by EA (Figure 10, ## *p* < 0.01, n = 6) and KO (Figure 10, ### *p* < 0.001), but not sham treatment (Figure 10, *** *p* < 0.001, n = 6). However, despite the expression in CB VI, there was no alteration in the levels of pPKA, pERK, and pJNK. They did not exhibit any significant changes among the five groups of mice.

#### 3.2.4. Role of TRPV1 and Its Associated Proteins in the Descending Pathways of CB VII and the Hypothalamus

**Figure 11:**
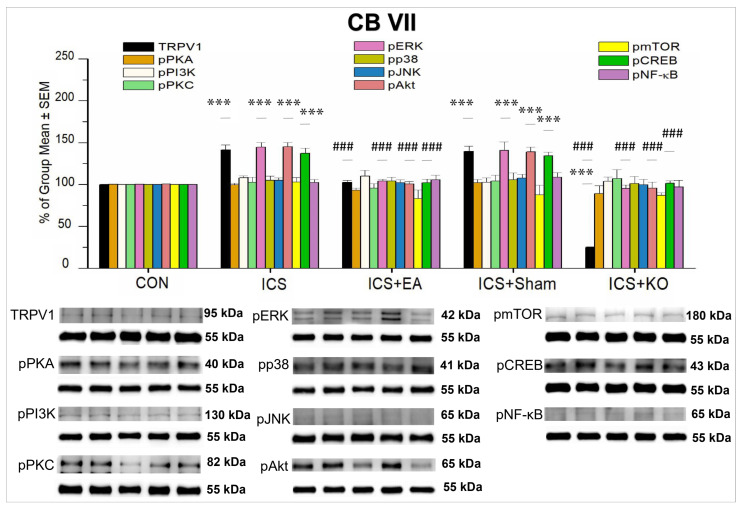
Percentage changes in the expression of TRPV1 and associated molecules in the CB VII of mice (%). The control group was set as 100% and served as a reference. The WB analysis included five lanes for each protein: CON, ICS, ICS + EA, ICS + Sham, and ICS + KO. The proteins observed were TRPV1, pPKA, pPI3K, pPKC, pAkt, pmTOR, pERK, pP38, pJNK, pCREB, and pNF-κB. α-Tubulin was used as an internal control protein. Statistical differences were analyzed using one-way ANOVA, followed by Tukey’s post hoc test. *** *p* < 0.001, compared to the CON group; ### *p* < 0.001, compared to the ICS group. n = 6.

Finally, the TRPV1 signaling pathway in CB VII and the hypothalamus was investigated by WB analysis (Table 4). In the CB VII brain regions, TRPV1, pERK, pAkt, and pCREB expression were increased in mice with FM induction (Figure 11, *** *p* < 0.001, n = 6) and was reversed by EA and KO (Figure 11, ### *p* < 0.001, n = 6) but not by sham EA (Figure 11, *** *p* < 0.001, n = 6). TRPV1 expression in KO mice was minimal compared to the CON mice (Figure 11, *** *p* < 0.001, n = 6). In contrast, we observed the expression of pPKA, pPI3K, pPKC, pP38, pJNK, pMTOR, and pNF-κB in the mouse CB VII, but no significant differences were found in their percentages across the five groups (Table 4).

**Figure 12:**
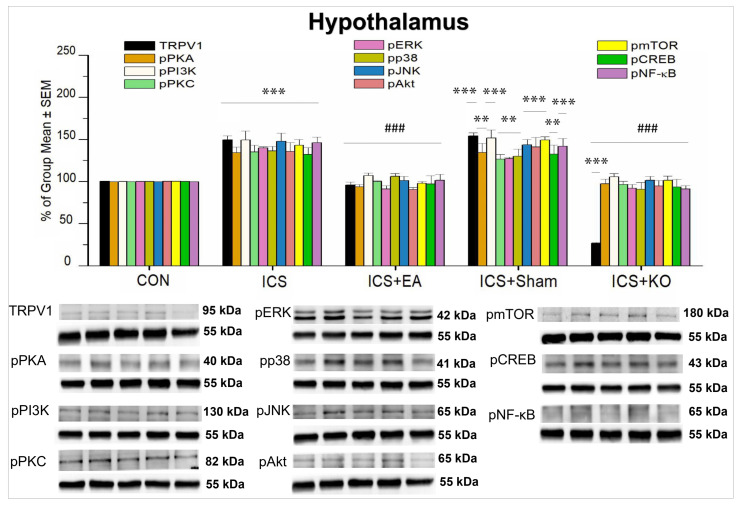
Percentage changes in the expression of TRPV1 and associated molecules in the hypothalamus of mice (%). The control group was set as 100% and served as a reference. The WB analysis included five lanes for each protein: CON, ICS, ICS + EA, ICS + Sham, and ICS + KO. The proteins observed were TRPV1, pPKA, pPI3K, pPKC, pAkt, pmTOR, pERK, pP38, pJNK, pCREB, and pNF-κB. α-Tubulin was used as an internal control protein. Statistical differences were analyzed using one-way ANOVA, followed by Tukey’s post hoc test. *** *p* < 0.001, ** *p* < 0.01, compared to the CON group; ### *p* < 0.001, compared to the ICS group. n = 6.

Similar to patterns in the thalamus, SSC, and mPFC, all proteins studied in the hypothalamus were potentiated after FM (Figure 12, *** *p* < 0.001, n = 6) and were significantly decreased by EA and KO (Figure 12, ### *p* < 0.001, n = 6). Sham EA treatment cannot alter the overexpression of TRPV1, pPI3K, pJNK, pAkt, pmTOR, and pNF-κB in mice after exposure to ICS (Figure 12, *** *p* < 0.001, n = 6). The increase in pPKA, pPKC, pERK, pP38, and pCREB was also unchanged after sham EA (Figure 12, ** *p* < 0.01, n = 6). TRPV1 expression in the KO group was significantly lower than that in the CON group (Figure 12, *** *p* < 0.001, n = 6).

## 4. Discussion

Fibromyalgia places a significant burden on patients, negatively affecting their health, productivity, and finances. This often leaves them unable to access treatment, creating a cycle of suffering. The average annual treatment cost for FM in the United States approached $40,000, largely due to the cost of prescribed FM medications [36]. Conversely, acupuncture is a viable and more affordable alternative for FM patients in the United States. Antidepressant medications may induce a range of side effects, including suicidal thoughts and life-threatening symptoms [37]. However, acupuncture has been shown to mitigate the risks associated with pharmaceutical treatments [38]. In clinical research, acupuncture demonstrated greater effectiveness in managing FM compared to fluoxetine after four weeks of treatment [39]. In addition, combining acupuncture with antidepressants resulted in a greater reduction in tender points and an improved pressure pain threshold after six months [40]. However, a meta-analysis emphasized that there was insufficient evidence to prove the effectiveness of acupuncture compared to sham acupuncture [38]. EA intervention in our study demonstrated a significant difference compared to the sham EA. This comparison excluded the placebo influence of psychological factors or expectations typically observed in humans. The benefits of EA suggest the potential clinical utility of supplementary treatment for FM patients.

Research demonstrated that EA is most effective when administered early in neurological and inflammatory conditions. Moreover, early intervention significantly accelerates recovery and enhances the quality of life in patients. However, many clinical studies categorized FM diagnosis into early (≤2 years), late (>2–7 years), and very late (>7 years) [41]. As the diagnostic criteria require symptoms to persist for at least 3 months, early treatment or prevention of FM is challenging [42]. Our current study examined the effect of acupuncture on chronic FM pain using the ICS model. In humans, FM is not explained by other disorders [42]. ICS reflects environmental factors of FM, with cold weather increasing pain and weather fluctuations worsening symptoms [43]. In mice, ICS induced chronic pain lasting over twelve days, compared to seven days of acute pain [35]. Additionally, depression- and anxiety-like behaviors were observed in the model, which improved following antidepressant treatment [44]. However, the model lacks evidence on fatigue and sleep disturbances. Furthermore, depressive-like behavior assessment was not addressed in this study to concentrate specifically on pain symptoms.

Our data showed that low-frequency EA at ST36 reliably reduced mechanical and thermal hyperalgesia in ICS-induced FM-like pain. Chronic hyperalgesia was observed in mice under ICS, with a significant reduction in pain threshold persisting until day 21. Hyperalgesia is defined as an exaggerated response to a stimulus that would typically cause pain [45]. Low-frequency transcutaneous electrical nerve stimulation (TENS) at low frequency can activate μ-opioid receptor to reduce primary mechanical hyperalgesia [46]. Additionally, TENS in acupoints effectively suppressed thermal hyperalgesia [47]. Acupuncture at 10 Hz and 100 Hz can alleviate chronic pain induced by acid saline injections [48].

FM is associated with neurochemical imbalance within the CNS. This dysregulation can exacerbate central and peripheral sensitization, leading to primary and secondary hyperalgesia. Treatments targeting the ascending and descending CNS pain pathway have shown effectiveness in managing FM symptoms [49]. The primary regions in the ascending pathway include the thalamus, SSC, and mPFC. The hippocampus is involved in both ascending and descending pain pathways, but its role is more prominent in the ascending pathway. Altered functional connectivity between the thalamus and inferior parietal lobule is associated with lower pain threshold to sensory stimuli in FM patients [50]. SSC activity increases with more intense painful stimuli and is involved in identifying the source of pain [51]. The mPFC plays a vital role in modulating pain, dampening pain-induced sympathetic activity, and mitigating facial expressions indicative of pain [52]. The CA1 region of the hippocampus processes pain signals to CA3 to form memories of chronic pain [53]. In chronic pain, the Hypothalamic–Pituitary–Adrenal axis becomes dysregulated, causing excessive cortisol production. This dysregulation can lead to fatigue, depression, and impaired immune function [54]. The posterior cerebellar lobes, including lobules V, VI, and VII, are involved in sensory processing and motor coordination [55]. In previous inflammatory pain experiments, protein expression changes were detected in CBV and CBVII with no significant changes in CBVI.

In the present study, pain-signaling proteins were detected in both afferent and efferent pain pathways. However, immunoblotting results revealed distinct changes during the chronic phase across studied brain regions. Chen et al. found that the PI3K/Akt/pmTOR/NF-kB signaling pathway was significantly elevated in the thalamus and SSC, but not in the DRG or SC, after 4 weeks of acid saline-induced FM [56]. ICS also potentiated Akt phosphorylation in the thalamus after a 5-day experiment [57]. The findings of the current study showed that pAkt was unaltered in the thalamus in any groups after 21 days of FM induction. However, the expression of the remaining proteins was significantly increased in the thalamus of the ICS group. In a 40-day model after ICS induction, the density of TRPV1 and other proteins was decreased in the mPFC, hippocampus, periaqueductal gray, and amygdala [58]. In a 14-day study, ICS increased the expression of pain signaling proteins in the descending pathway brain regions, including the hypothalamus, CB V, CB VI, and CB VII [59]. In our study, potentiation was observed in TRPV1 and its related downstream molecules in the SSC, mPFC, and hypothalamus. Conversely, data showed that the hippocampus exhibited upregulation in the density of TRPV1, pPI3K, pJNK, pmTOR, and pNF-κB. Regarding the descending pain pathway, including CB V, VI, and VII, the density of TRPV1 and several of its downstream proteins were potentiated in the ICS group. In a previous study, TRPV1 and its related proteins were unchanged in CB VI following CFA-induced inflammatory pain [60].

TRPV1 activation triggers extensive hyperphosphorylation of kinases. These proteins are expressed in neuronal cells, microglia, and astrocytes, which are critical for the CNS response to injury and inflammatory pain. EA suppressed the expression of glial cell markers in the PNS and CNS. TRPV1 and μ-opioid receptors are involved in the regulation of β-arrestin 2 to regulate inflammatory responses. Therefore, treatments targeting both the μ-opioid and the TRPV1 receptors are believed to be beneficial in eliminating pain and preventing opioid addiction [61]. Adenosine levels were potentiated while substance P and inflammatory agents such as IL-1, IL-6, and TNF-α were decreased by EA at the ST36 acupoint [62]. Moreover, adenosine correction relates to the modulation of Nav1.8, COX-2, and pPKCε [63]. The kinases PI3K/Akt play a crucial role in modulating pain through the effect on mediated inflammation and reducing cytokines such as TNF-α and IL-17 [64]. EA inhibited the phosphorylation of PI3K and Akt, which activated the inflammatory response, reducing hyperalgesia in mice injected with carrageenan [65]. TRPV1 can stimulate anandamide (AEA) synthesis, which in turn activates PKC [66]. Flow cytometry experiments revealed that pERK, p38, and pJNK are co-phosphorylated after a proinflammatory injection, implying a role in pain signaling [67]. Our findings suggest that pJNK and pP38 have been associated with increased sensitivity to toxic stimuli in chronic pain. cAMP (PKA) increases intracellular calcium transients through phosphorylation, leading to neuronal sensitization. pPKA can activate various ion channels, including voltage-gated calcium channels such as TRPV1 and hyperpolarization-activated cyclic nucleotide-gated channels. The activation of these channels facilitated calcium influx into the cell, either directly or indirectly. As a result, intracellular calcium levels were increased [68]. Previously, the PKA/CREB signaling pathway was renowned for its association with the mechanisms underlying inflammatory pain.

Our data demonstrated potentiation of TRPV1 and its associated proteins in the thalamus, SSC, mPFC, hippocampus, CB V, VI, VII, and hypothalamus of adult mice. This overexpression was reduced by EA treatment and in TRPV1 KO mice, but not by sham EA treatment. Previous studies indicated that these brain regions are important to pain signaling. In this study, the expression of TRPV1 and other proteins after ICS differed in each region, especially in the hippocampus, CB V, VI, and VII. The results eliminated the placebo effect by employing a sham EA group without de-qi sensation. Deletion of the TRPV1 gene in mice resulted in the absence of physical hypersensitivity. Further investigation is needed, particularly focusing on brain regions to determine protein localization. Additionally, a comparison with other common medicines is necessary. Its relevance to clinical application, however, remains unaddressed.

## 5. Conclusions

**Figure 13:**
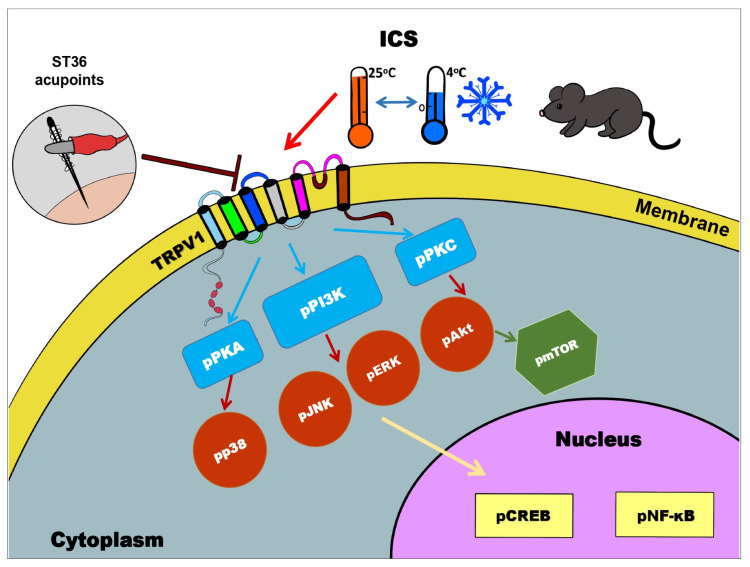
Schematic mechanism of EA in FM treatment via TRPV1 and related downstream molecules.

In summary, our findings demonstrate that EA effectively alleviates mechanical and thermal hypersensitivity associated with long-term pain. This heightened sensitivity to noxious stimuli is associated with the role of TRPV1 and its downstream signaling molecules in the CNS. Notably, the presence of TRPV1 and associated pain signaling pathways contributes to central sensitization in brain regions, including the thalamus, SSC, mPFC, hippocampus, CB V, VI, VII, and hypothalamus (Figure 13).

## Figures and Tables

**Table 1:** Comparison of protein percentages (%) in the thalamus and SSC across five groups (experiment WB). The control group was set as 100% and served as a reference. TRPV1: Transient Receptor Potential Vanilloid 1, pPKA: phosphorylated Protein Kinase A, pPI3K: phosphorylated Phosphoinositide 3-Kinase, pPKC: phosphorylated Protein Kinase C, pERK: phosphorylated Extracellular Signal-Regulated Kinase, pP38: phosphorylated P38 Mitogen-Activated Protein Kinase, pJNK: phosphorylated c-Jun N-terminal Kinase, pAkt: phosphorylated Protein Kinase B, pmTOR: phosphorylated Mechanistic Target of Rapamycin, pCREB: phosphorylated cAMP Response Element-Binding Protein, pNFκB: phosphorylated Nuclear Factor Kappa B. Statistical differences were analyzed using repeated one-way ANOVA and double-checked using Tukey’s post hoc test. *** denotes a significant difference, compared to the CON group (*p* < 0.001); ### denotes a significant difference, compared to the ICS group (*p* < 0.001).

Brain Region	Protein	CON (%)	ICS (%)	ICS + EA (%)	ICS + Sham (%)	ICS + KO (%)
Thalamus	TRPV1	102.15 ± 1.89	157.86 ± 6.97 ***	105.93 ± 3.88 ###	152.95 ± 6.07 ***	16.36 ± 1.95 ***,###
	pPKA	100.08 ± 0.27	150.35 ± 6.62 ***	104.55 ± 2.21 ###	146.65 ± 4.52 ***	103.78 ± 3.22 ###
	pPI3K	99.6 ± 0.29	146.81 ± 5.05 ***	102.15 ± 4.99 ###	146.03 ± 4.89 ***	103.93 ± 1.75 ###
	pPKC	99.23 ± 0.57	161.5 ± 4.17 ***	101.8 ± 2.01 ###	160.85 ± 5.11 ***	98.98 ± 3.92 ###
	pERK	99.83 ± 0.39	171.5 ± 5.47 ***	104.63 ± 5.52 ###	161.46 ± 5.63 ***	88.85 ± 7.54 ###
	pP38	99.96 ± 0.09	138.4 ± 3.43 ***	100.45 ± 3.15 ###	134.06 ± 3.53 ***	87.35 ± 5.57 ###
	pJNK	99.93 ± 0.26	161.23 ± 7.35 ***	103.16 ± 1.40 ###	161.45 ± 6.88 ***	99.38 ± 1.66 ###
	pAkt	99.99 ± 0.1	98.21 ± 1.07	99.89 ± 2.89	98.28 ± 1.81	96.27 ± 2.46
	pmTOR	99.25 ± 0.79	159.91 ± 8.01 ***	109.08 ± 3.84 ###	161.1 ± 6.55 ***	107.98 ± 4.24 ###
	pCREB	100.3 ± 0.47	155.05 ± 6.04 ***	109.26 ± 2.99 ###	155.58 ± 7.33 ***	99.46 ± 5.18 ###
	pNF-κB	100.2 ± 0.21	150.11 ± 5.26 ***	100.36 ± 2.46 ###	147.85 ± 7.26 ***	100.2 ± 3.07 ###
SSC	TRPV1	100.35 ± 0.48	158.3 ± 2.74 ***	97.05 ± 5.40 ###	144.61 ± 6.07 ***	15.66 ± 2.37 ***,###
	pPKA	99.93 ± 0.02	137.63 ± 4.33 ***	105.65 ± 3.51 ###	136.2 ± 5.64 ***	89.08 ± 5.16 ###
	pPI3K	99.85 ± 0.73	144.58 ± 3.20 ***	104.41 ± 5.29 ###	143.13 ± 4.12 ***	84.93 ± 5.33 ###
	pPKC	101.1 ± 1.13	147.81 ± 4.85 ***	114.28 ± 3.54 ###	143.05 ± 5.99 ***	103.31 ± 4.45 ###
	pERK	100.05 ± 0.02	154.83 ± 4.58 ***	111.31 ± 3.22 ###	160.88 ± 11.01 ***	102.7 ± 10.15 ###
	pP38	99.18 ± 0.94	137.93 ± 3.39 ***	107.58 ± 2.18 ###	139.9 ± 2.84 ***	104.28 ± 5.28 ###
	pJNK	99.85 ± 0.21	129.01 ± 1.38 ***	102.68 ± 2.24 ###	127.35 ± 3.50 ***	101.58 ± 6.80 ###
	pAkt	100.33 ± 0.36	137.23 ± 4.43 ***	108.25 ± 2.20 ###	132.66 ± 3.00 ***	100.51 ± 4.24 ###
	pmTOR	99.45 ± 0.37	148.33 ± 5.58 ***	104.78 ± 7.55 ###	144.55 ± 7.60 ***	103.71 ± 9.28 ###
	pCREB	99.66 ± 0.26	151.1 ± 3.05 ***	109.63 ± 4.50 ###	150.9 ± 4.76 ***	94.28 ± 5.82 ###
	pNF-κB	99.51 ± 0.11	136.95 ± 5.31 ***	105.93 ± 3.71 ###	133.23 ± 4.97 ***	104.2 ± 3.71 ###

**Table 2:** Comparison of protein percentages (%) in mPFC and hippocampus (Hippo) across five groups (experiment WB). The control group was set as 100% and served as a reference. TRPV1: Transient Receptor Potential Vanilloid 1, pPKA: phosphorylated Protein Kinase A, pPI3K: phosphorylated Phosphoinositide 3-Kinase, pPKC: phosphorylated Protein Kinase C, pERK: phosphorylated Extracellular Signal-Regulated Kinase, pP38: phosphorylated P38 Mitogen-Activated Protein Kinase, pJNK: phosphorylated c-Jun N-terminal Kinase, pAkt: phosphorylated Protein Kinase B, pmTOR: phosphorylated Mechanistic Target of Rapamycin, pCREB: phosphorylated cAMP Response Element-Binding Protein, pNFκB: phosphorylated Nuclear Factor Kappa B. Statistical differences were analyzed using one-way ANOVA and Tukey’s post hoc test. *** Denotes a significant difference in comparison to the Control group (*p* < 0.001), ### denotes a significant difference compared to the ICS group (*p* < 0.001).

Brain Region	Protein	CON (%)	ICS (%)	ICS + EA (%)	ICS + Sham (%)	ICS + KO (%)
mPFC	TRPV1	100.1 ± 0.67	160.7 ± 4.97 ***	103.86 ± 2.27 ###	161.23 ± 8.32 ***	7.76 ± 1.83 ***,###
	pPKA	100.05 ± 0.21	155.01 ± 2.92 ***	104.98 ± 5.93 ###	145.48 ± 5.29 ***	103.36 ± 4.87 ###
	pPI3K	99.88 ± 0.17	141.9 ± 5.20 ***	95.23 ± 4.23 ###	143.36 ± 5.24 ***	99.28 ± 2.39 ###
	pPKC	99.83 ± 0.12	151.26 ± 2.86 ***	96.85 ± 8.43 ###	146.53 ± 6.26 ***	97.03 ± 4.53 ###
	pERK	100.21 ± 0.15	150.43 ± 6.95 ***	100.06 ± 4.13 ###	139.71 ± 3.91 ***	79.58 ± 7.68 ###
	pP38	99.96 ± 0.08	147.21 ± 3.82 ***	103.76 ± 3.53 ###	151 ± 3.45 ***	99.36 ± 2.99 ###
	pJNK	100.35 ± 0.36	158.16 ± 4.40 ***	106.43 ± 4.76 ###	150.3 ± 6.06 ***	99.43 ± 3.18 ###
	pAkt	100.03 ± 0.26	164.43 ± 3.10 ***	111.26 ± 3.38 ###	158.01 ± 3.88 ***	106.21 ± 3.55 ###
	pmTOR	100.03 ± 0.55	154.98 ± 5.50 ***	101.8 ± 4.39 ###	161.55 ± 5.82 ***	98.1 ± 2.73 ###
	pCREB	99.88 ± 0.24	162.76 ± 6.06 ***	112.65 ± 4.11 ###	166.33 ± 3.20 ***	103.65 ± 5.40 ###
	pNF-κB	99.85 ± 0.26	175.91 ± 3.40 ***	103.48 ± 4.86 ###	172.95 ± 6.59 ***	88.71 ± 3.61 ###
Hippo	TRPV1	99.48 ± 0.62	136.30 ± 4.76 ***	100.17 ± 3.05 ###	137.92 ± 5.22 ***	24.26 ± 1.46 ***,###
	*pPKA*	99.91 ± 0.21	99.52 ± 5.92	108.47 ± 9.40	97.59 ± 2.25	96.79 ± 4.95
	pPI3K	100.04 ± 0.23	133.89 ± 9.05 ***	97.83 ± 0.41 ###	132.04 ± 5.01 ***	97.28 ± 2.88 ###
	*pPKC*	99.82 ± 0.19	91.78 ± 2.36	107.74 ± 9.82	96.65 ± 4.74	104.09 ± 5.75
	*pERK*	100.1 ± 0.17	94.84 ± 2.59	98.72 ± 8.06	94.98 ± 7.46	89.71 ± 5.66
	*pP38*	99.71 ± 0.10	101.03 ± 5.53	97.75 ± 3.40	99.9 ± 3.30	89.29 ± 3.74
	pJNK	99.94 ± 0.31	130.89 ± 2.32 ***	102.96 ± 1.46 ###	129.86 ± 3.90 ***	105.41 ± 1.25 ###
	*pAkt*	100.01 ± 0.14	100.31 ± 7.46	99.98 ± 6.39	103.21 ± 8.92	102.95 ± 4.34
	pmTOR	100.22 ± 0.14	144.94 ± 7.76 ***	102.91 ± 3.18 ###	148.85 ± 8.09 ***	103.37 ± 2.19 ###
	*pCREB*	100.11 ± 0.14	95.32 ± 9.37	101.34 ± 4.78	105.71 ± 5.42	101.24 ± 8.67
	pNF-κB	99.73 ± 0.07	125.95 ± 4.22 ***	96.58 ± 4.34 ###	122.40 ± 3.17 ***	97.44 ± 5.67 ###

**Table 3:** Comparison of protein percentages (%) in CB V and CB VI across experimental groups (experiment WB). The control group was set as 100% and served as a reference. TRPV1: Transient Receptor Potential Vanilloid 1, pPKA: phosphorylated Protein Kinase A, pPI3K: phosphorylated Phosphoinositide 3-Kinase, pPKC: phosphorylated Protein Kinase C, pERK: phosphorylated Extracellular Signal-Regulated Kinase, pP38: phosphorylated P38 Mitogen-Activated Protein Kinase, pJNK: phosphorylated c-Jun N-terminal Kinase, pAkt: phosphorylated Protein Kinase B, pmTOR: phosphorylated Mechanistic Target of Rapamycin, pCREB: phosphorylated cAMP Response Element-Binding Protein, pNFκB: phosphorylated Nuclear Factor Kappa B. Statistical differences were analyzed using one-way ANOVA and Tukey’s post hoc test. **, *** Denotes a significant difference in comparison to the control group (*p* < 0.01, or *p* < 0.001), ##, ### denotes a significant difference compared to the ICS group (*p* < 0.01, or *p* < 0.001).

Brain Region	Protein	CON (%)	ICS (%)	ICS + EA (%)	ICS + Sham (%)	ICS + KO (%)
**CBV**	TRPV1	99.55 ± 0.63	155.29 ± 7.73 ***	100.27 ± 3.01 ###	151.11 ± 8.42 ***	26.15 ± 1.14 ***, ###
	pPKA	100.13 ± 0.18	157.92 ± 5.49 ***	110.42 ± 6.45 ###	170.84 ± 12.04 ***	99.08 ± 13.15 ###
	pPI3K	100.06 ± 0.14	149.3 ± 13.09 ***	99.40 ± 2.94 ###	144.25 ± 4.19 ***	87.76 ± 7.18 ###
	pPKC	100.16 ± 0.20	128.77 ± 4.81 ***	103.73 ± 4.51 ###	123.02 ± 4.37 ***	101.65 ± 2.64 ###
	*pERK*	100.15 ± 0.14	91.12 ± 19.02	60.59 ± 26.65	55.53 ± 16.47	61.16 ± 9.71
	*pP38*	100.02 ± 0.05	101.72 ± 2.37	100.43 ± 4.01	103.72 ± 4.75	99.41 ± 8.32
	*pJNK*	99.91 ± 0.16	114.17 ± 14.68	115.47 ± 16.12	111.28 ± 7.17	101.21 ± 8.90
	pAkt	100.01 ± 0.10	125.44 ± 3.65 ***	86.69 ± 5.20 ###	125.10 ± 5.47 **	83.84 ± 7.89 ##
	pmTOR	100.39 ± 0.59	159.69 ± 3.63 ***	97.58 ± 6.11 ###	139.32 ± 8.01 **	86.21 ± 16.80 ##
	*pCREB*	100.03 ± 0.20	109.93 ± 3.81	97.09 ± 9.79	107.53 ± 6.11	107.99 ± 7.97
	pNF-κB	99.59 ± 0.23	155.50 ± 5.07 ***	110.66 ± 6.02 ###	146.87 ± 9.93 **	105.09 ± 9.37 ##
**CBVI**	TRPV1	100.51 ± 0.32	136.53 ± 1.39 ***	102.45 ± 3.80 ###	140.47 ± 3.97 ***	27.01 ± 1.33 ***,###
	*pPKA*	100.26 ± 0.08	107.88 ± 2.62	106.86 ± 6.79	104.00 ± 5.16	94.94 ± 7.84
	pPI3K	100.25 ± 0.29	138.62 ± 8.61 ***	111.33 ± 6.13 ##	137.87 ± 8.27 ***	94.62 ± 4.96 ###
	pPKC	100.16 ± 0.17	135.61 ± 4.22 ***	104.09 ± 5.54 ###	128.54 ± 8.08 ***	92.71 ± 6.45 ###
	*pERK*	100.00 ± 0.11	96.98 ± 8.55	83.15 ± 21.57	89.87 ± 15.50	83.60 ± 13.75
	pP38	100.31 ± 0.41	150.73 ± 2.27 ***	117.84 ± 6.24 ##	145.31 ± 7.01 ***	88.91 ± 10.01 ###
	*pJNK*	99.92 ± 0.11	113.02 ± 9.09	104.24 ± 6.54	109.31 ± 3.45	103.61 ± 4.41
	pAkt	99.77 ± 0.28	139.54 ± 1.07 ***	105.03 ± 0.76 ###	133.46 ± 3.68 ***	94.61 ± 2.25 ###
	pmTOR	100.27 ± 0.45	152.34 ± 11.70 ***	112.65 ± 6.11 ##	145.04 ± 8.41 ***	91.17 ± 6.05 ###
	pCREB	99.65 ± 0.17	139.49 ± 7.90 ***	108.45 ± 3.28 ###	137.89 ± 7.68	80.43 ± 9.71 ###
	pNF-κB	99.51 ± 0.30	131.25 ± 3.13 ***	101.24 ± 1.64 ###	129.16 ± 4.32 ***	100.84 ± 4.26 ###

**Table 4:** Comparison of protein percentages in CB VII and the hypothalamus (Hypo) across experimental groups (Experiment WB). The control group was set as 100% and served as a reference. TRPV1: Transient Receptor Potential Vanilloid 1, pPKA: phosphorylated Protein Kinase A, pPI3K: phosphorylated Phosphoinositide 3-Kinase, pPKC: phosphorylated Protein Kinase C, pERK: phosphorylated Extracellular Signal-Regulated Kinase, pP38: phosphorylated P38 Mitogen-Activated Protein Kinase, pJNK: phosphorylated c-Jun N-terminal Kinase, pAkt: phosphorylated Protein Kinase B, pmTOR: phosphorylated Mechanistic Target of Rapamycin, pCREB: phosphorylated cAMP Response Element-Binding Protein, pNFκB: phosphorylated Nuclear Factor Kappa B. Statistical differences were analyzed using one-way ANOVA and Tukey’s post hoc test. **, *** Denotes a significant difference in comparison to the control group (*p* < 0.01, or *p* < 0.001), ### denotes a significant difference compared to the ICS group (*p* < 0.001).

Brain Region	Protein	CON (%)	ICS (%)	ICS + EA (%)	ICS + Sham (%)	ICS + KO (%)
CB VII	TRPV1	99.93 ± 0.17	141.34 ± 5.88 ***	102.52 ± 2.45 ###	139.48 ± 6.30 ***	25.09 ± 0.60 ***,###
	*pPKA*	100.33 ± 0.11	99.76 ± 1.77	93.21 ± 2.25	101.93 ± 3.98	89.06 ± 9.31
	*pPI3K*	99.85 ± 0.13	107.94 ± 2.46	110.17 ± 6.51	102.75 ± 5.24	103.7 ± 5.14
	*pPKC*	99.92 ± 0.15	102.53 ± 6.12	95.54 ± 5.30	104.14 ± 7.19	107.17 ± 10.55
	pERK	100.38 ± 0.20	144.46 ± 5.72 ***	104.02 ± 2.33 ###	140.84 ± 9.87 ***	95.25 ± 4.09 ###
	*pP38*	100.10 ± 0.09	105.01 ± 5.06	104.11 ± 4.47	105.45 ± 8.43	101.31 ± 8.36
	*pJNK*	100.08 ± 0.07	105.15 ± 2.68	102.04 ± 3.59	107.55 ± 4.53	99.83 ± 6.21
	pAkt	100.48 ± 0.12	145.28 ± 4.80 ***	100.46 ± 3.19 ###	138.80 ± 5.83 ***	95.67 ± 7.27 ###
	*pmTOR*	99.98 ± 0.18	102.96 ± 5.18	83.08 ± 10.47	87.59 ± 11.64	86.95 ± 3.43
	pCREB	99.99 ± 0.14	137.2 ± 6.15 ***	101.94 ± 4.66 ###	134.16 ± 4.45 ***	101.33 ± 3.11 ###
	*pNF-κB*	99.94 ± 0.27	102.36 ± 3.44	105.52 ± 5.81	108.63 ± 5.65	97.12 ± 7.78
Hypo	TRPV1	100.29 ± 0.30	149.64 ± 4.75 ***	95.76 ± 3.73 ###	154.23 ± 3.62 ***	26.63 ± 1.06 ***,###
	pPKA	99.66 ± 0.31	134.21 ± 6.86 ***	93.72 ± 2.82 ###	134.42 ± 11.03 **	97.37 ± 5.69 ###
	pPI3K	99.98 ± 0.13	149.33 ± 10.77 ***	107.09 ± 2.82 ###	151.50 ± 9.49 ***	105.61 ± 3.86 ###
	pPKC	99.80 ± 0.22	135.23 ± 7.79 ***	99.99 ± 0.78 ###	126.46 ± 5.61 **	96.50 ± 3.89 ###
	pERK	99.94 ± 0.36	139.68 ± 1.61 ***	91.10 ± 4.01 ###	127.05 ± 1.86 **	91.93 ± 4.50 ###
	pP38	100.09 ± 0.32	136.36 ± 5.23 ***	106.08 ± 3.30 ###	130.19 ± 8.19 **	90.82 ± 7.88 ###
	pJNK	99.77 ± 0.21	147.87 ± 9.57 ***	101.03 ± 5.02 ###	143.79 ± 6.08 ***	101.47 ± 4.74 ###
	pAkt	100.20 ± 0.34	135.58 ± 10.87 ***	90.54 ± 2.48 ###	140.86 ± 11.44 ***	94.64 ± 6.46 ###
	pmTOR	100.31 ± 0.35	143.00 ± 6.88 ***	97.94 ± 1.94 ###	149.22 ± 3.99 ***	101.44 ± 4.92 ###
	pCREB	100.04 ± 0.15	132.23 ± 8.03 ***	97.12 ± 9.66 ###	132.37 ± 10.91 **	93.35 ± 9.40 ###
	pNF-κB	99.84 ± 0.13	146.15 ± 6.64 ***	101.33 ± 7.22 ###	142.00 ± 9.09 ***	91.07 ± 3.81 ###

## Data Availability

The original contributions presented in this study are included in the article. Further inquiries can be directed to the corresponding author.

## Abbreviations

TRPV1	Transient Receptor Potential Vanilloid 1
PKA	Protein Kinase A
PI3K	Phosphoinositide 3-Kinase
Akt	Protein Kinase B
JNK	c-Jun N-terminal Kinase
mTOR	Mammalian Target of Rapamycin
ERK1/2	Extracellular Signal-Regulated Kinase 1/2
P38	P38 Mitogen-Activated Protein Kinase
NF-κB	Nuclear Factor kappa-light-chain-enhancer of activated B cells
CREB	cAMP Response Element-Binding Protein
PKC	Protein Kinase C
